# Integrated analysis of gut microbiome and its metabolites in ACE2-knockout and ACE2-overexpressed mice

**DOI:** 10.3389/fcimb.2024.1404678

**Published:** 2024-07-17

**Authors:** Lini Song, Wenyi Ji, Xi Cao

**Affiliations:** Beijing Diabetes Institute, Beijing Key Laboratory of Diabetes Research and Care, Department of Endocrinology, Beijing Tongren Hospital, Capital Medical University, Beijing, China

**Keywords:** renin angiotensin system, metabolic syndrome, angiotensin-converting enzyme 2, gut microbiota, 16S rRNA gene sequencing

## Abstract

**Background:**

Aberrant activation of the classic renin–angiotensin system (RAS) and intestinal micro dysbiosis adversely affect insulin resistance (IR), dyslipidemia, and other metabolic syndrome markers. However, the action of angiotensin-converting enzyme 2 (ACE2) and gut health in systemic homeostasis vary, and their interaction is not completely understood.

**Methods:**

We adopted a combinatory approach of metabolomics and fecal 16S rRNA analysis to investigate gut microbiota and metabolite in two different mouse models, *ACE2* knockout (*ACE2* KO) mice and the ACE2-overexpressing obese mice.

**Results:**

16S rRNA gene sequencing revealed that ACE2 influences microbial community composition and function, and ACE2 KO mice had increased *Deferribacteres*, *Alcaligenaceae*, *Parasutterella*, *Catenibacterium*, and *Anaerotruncus*, with decreased short-chain fatty acid (SCFA)-producing bacteria (*Marvinbryantia* and *Alistipes*). In contrast, ACE2-overexpressed mice exhibited increased anti-inflammatory probiotic (*Oscillospiraceae*, *Marinifilaceae*, and *Bifidobacteriaceae*) and SCFA-producing microbes (*Rikenellaceae, Muribaculaceae*, *Ruminococcaceae*, *Odoribacter*, and *Alistipes*) and decreased *Firmicutes/Bacteroidetes*, *Lactobacillaceae*, *Erysipelotrichaceae*, and *Lachnospiraceae*. Metabolome analysis indicated differential metabolites in *ACE2* KO and ACE2-overexpression mice, especially the glucolipid metabolism-related compounds. Furthermore, correlation analysis between gut microbiota and metabolites showed a dynamic mutual influence affecting host health.

**Conclusion:**

Our study confirms for the first time a significant association between *ACE2* status and gut microbiome and metabolome profiles, providing a novel mechanism for the positive effect of ACE2 on energy homeostasis.

## 1 Introduction

Metabolic syndrome (MetS) is a complex group of clinical symptoms characterized by hyperglycemia, hypercholesterolemia, central obesity, and hypertension, increasing the risk of type 2 diabetes (T2DM), cardiovascular disease, and hepatic diseases (Lemieux and Després, 2020). Along with the genetics and environmental factors, other contributors such as aberrant activation of the renin–angiotensin system (RAS) and imbalanced gut microbiota can cause host energy metabolism disorders, leading to insulin resistance (IR), dyslipidemia, and other hallmarks of MetS (Putnam et al., 2012; Thomas et al., 2022). However, the interrelationship between RAS and the intestinal microenvironment in systemic homeostasis varies and is poorly understood.

RAS consists of the classic angiotensin-converting enzyme (ACE)/angiotensin (Ang) II pathway and the novel ACE2/Ang-(1–7) pathway, which has recently gained importance in the context of glucose and lipid metabolism, beyond its role in cardiovascular homeostasis. With the advanced recognition of local RAS, emerging studies indicate that the complete range of RAS components is expressed and active throughout the entire gastrointestinal (GI) tract (Zamolodchikova et al., 2016). Tung Po Wong and associates have confirmed that in enterocytes, RAS showed downregulation of angiotensin type 1 receptor (AT1), AT2, and ACE, but upregulation of the ACE2/Ang-(1-7)/Mas receptor axis in the streptozotocin-induced diabetes model. Unlike the opposing actions of Ang II and Ang-(1–7) in other tissues, both peptides favorably influence blood glucose by blocking sodium-glucose cotransporter 1, namely SGLT1, and glucose transporter (GLUT) mediated uptake in the intestine (Wong et al., 2009, 2012). However, immunofluorescence assay exhibited a loss of ACE2 in the small intestine of *Akita* mice (a model of T1DM), and ACE2 deletion aggravates gut barrier disruption and hyperglycemia (Prasad et al., 2023).

The gut microbiome, often referred to as the “second genome” of humans, plays crucial physiological roles. Changes in the gut microbiome composition and structure or its metabolites, such as short-chain fatty acids (SCFAs) and bile acid, are associated with fat accumulation and impaired glycemic control (Fujisaka et al., 2023). In addition, there is a close link between the gut microbiota and the RAS, particularly ACE2. Emerging evidence reports that the GI tract is a route of entry for severe acute respiratory syndrome coronavirus 2 (SARS-CoV-2) via ACE2, resulting in enteric ACE2 dysregulation, and followed by diarrhea and gut dysbiosis (Juthi et al., 2023). Conversely, gut bacterial species and SCFA may also mediate intestinal ACE2 expression (Brown et al., 2022).

Our previous research confirmed that *ACE2* knockout mice showed a MetS-like state characterized by IR and hepatic steatosis, while ACE2 upregulation may reverse these effects by improving stress state, mitochondrial dysfunction, and insulin receptor substrate -1/protein kinase B/Adenosine 5’-monophosphate (AMP)-activated protein kinas, namely IRS-1/Akt/AMPK signaling (Song et al., 2021). On the other hand, *ACE2* deficiency results in lower body weight, which may be related to intestinal inflammation and impaired neutral essential amino acid absorption due to the decreased ACE2:B0AT1 complex (Hashimoto et al., 2012). Moreover, relevant studies have shown that *ACE2* deficiency disrupted the microbiome and gut-vascular integrity by depleting angiogenic components of the bone marrow in diabetes, potentially causing bacterial translocation (Duan et al., 2018). However, there are no data yet that show that changes in ACE2 levels directly alter gut bacteria and thereby alter fecal metabolites, which may cause metabolic disorder. In this study, we explored cutting-edge evidence on the interactions between ACE2 and the intestinal microenvironment, the main microbial changes related to glucolipid metabolism.

## 2 Materials and methods

### 2.1 Animals

The present study utilized ACE2 knockout (*ACE2* KO, *ACE2* ^-/y^) mice, as previously described (Niu et al., 2008). Twelve-week-old male *ACE2* KO mice and their wild-type littermates (WT) were fed a normal chow diet (13.5% calories from fat; Vital River Laboratory Animal Technology Co., Ltd, Beijing, China). A polymerase chain reaction (PCR) approach was used to confirm the *ACE2* KO mice with genomic DNA extracted from tail biopsies. Six-week-old male C57BL/6J mice, purchased from Vital River Laboratory Animal Technology (Beijing, China), were used to induce obesity by feeding them a high-fat diet (HFD; 60 kcal% fat) (Research Diets, New Brunswick, NJ, USA) for 8 weeks. The mice were then randomly divided into control and ACE2-overexpression groups. Adenovirus coding rat ACE2 (Ad-ACE2) and the control green fluorescent protein (Ad-GFP) were respectively injected into the obese mice via the tail vein [5 × 10^8^ particle-forming units (pfu) in 100 μL of saline]. The animals were used on the 6th day post-virus injection. All mice were housed in standard animal laboratories with constant temperature and humidity, in an artificial 12-h light/dark cycle, and given free access to food and water. All animal care and experimental protocols were approved by the Ethics Committee of Animal Research at Beijing Tongren Hospital, Capital Medical University, Beijing, China.

### 2.2 Fecal DNA extraction and amplicon generation

The experimental mice were housed in individual cages to minimize fecal contamination. Approximately 2 g of mouse stool samples was collected, placed in sterile DNA/RNA-free tubes, frozen with liquid nitrogen, and preserved in an −80°C freezer until further analyses. Total genome DNA from the samples was extracted using the Cetyltrimethylammonium bromide (CTAB) method. DNA concentration and purity were monitored on 1% agarose gels, and 10 ng of template DNA was chosen for subsequent amplification. The 16S rRNA genes of distinct V4 hypervariable region were amplified using specific the primer 515F (5′-GTGCCAGCMGCCGCGGTAA-3′) and 806R (5′-GGACTACHVGGGTWTCTAAT-3′) with a barcode. Phusion^®^ High-Fidelity PCR Master Mix (New England Biolabs, Ipswich, MA, UK) and efficient high-fidelity enzymes were used to ensure amplification efficiency and accuracy. Thermal conditions consisted of an initial denaturation at 98°C for 1 min, followed by 30 cycles of denaturation at 98°C for 10 s, annealing at 50°C for 30 s, and elongation at 72°C for 30 s, with a final extension of 5 min at 72°C. The PCR products were detected by electrophoresis and purified with the Qiagen Gel Extraction Kit (Qiagen, Germany).

### 2.3 Expression analyses

The real-time PCR was performed on the Light Cycler 480 real‐time PCR system (Roche, Basel, Switzerland) using the SYBR Green PCR Master Mix kit (Applied Biosystems, Foster City, CA). The primer sequences are as follows: β-actin, Forward primer 5′-AGTGTGACGTTGACATCCGTA-3′, Reverse primer 5′-GCCAGAGCAGTAATCTCCTTCT-3′; ACE2, Forward primer 5′-TCCAGACTCCGATCATCAAGC-3′, Reverse primer 5′-TGCTCATGGTGTTCAGAATTGT-3′.

### 2.4 16S rRNA sequencing and analysis

Sequencing work was performed by the NovelBio Bio-Pharm technology company using an Illumina MiSeq platform. Raw sequence data were obtained and paired-end reads were merged using FASTA36.3.6. Raw tags were processed by the Quantitative Insights into Microbial Ecology pipeline (QIIME, v1.9.0, http://qiime.org) to obtain high-quality clean tags. In summary, sequences with ambiguous bases, a length of less than 150 bp, or a Phred quality score <20 were deleted. The chimera sequences were identified and removed using the UCHIME algorithm. Finally, all effective tags were clustered using the Uparse algorithm, and sequences with ≥97% similarity were assigned to the same operational taxonomic units (OTUs). A representative sequence of each OUT was screened to annotate taxonomic assignments using the Silva Database (SSU128, https://www.arb-silva.de) based on the Mothur algorithm. Normalized OTU abundance information was used for further alpha and beta diversity with the QIIME sequence analysis package. The relative abundance and proportion of species at different classification levels (phylum, class, order, family, genus, and species) of each sample were presented and analyzed using a microbial community barplot based on species annotation. Furthermore, biomarkers and metabolic functional traits of the microbiome were analyzed using LDA Effect Size (LEfSe) and Phylogenetic Investigation of Communities by Reconstruction of Unobserved States (PICRUS).

### 2.5 Metagenomic sequencing and analysis

The extracted, purified DNA of each sample was used to construct the sequencing library using the NEBNext^®^ Ultra™ DNA Library Prep Kit for Illumina (NEB, USA) following the manufacturer’s recommendations. Briefly, genomic DNA was randomly fragmented by restriction enzyme to a size of 350 bp. Then, the DNA fragments were end-polished, A-tailed, and ligated with the full-length adaptor for Illumina sequencing, followed by PCR amplification. The prepared libraries were evaluated for size distribution using Agilent 2100 Bioanalyzer (Agilent Technologies, Santa Clara, California, USA) and quantified using real-time PCR. Whole-genome sequencing was performed on an Illumina HiSeq platform. The sequencing raw data were preprocessed using Readfq (V8, https://github.com/cjfields/readfq) and Bowtie2.2.4 software (http://bowtiebio.sourceforge.net/bowtie2/index.shtml) to remove the reads that were unqualified and of host origin. The SOAPdenovo software (V2.04, http://soap.genomics.org.cn/soapdenovo.html) was employed for the single sample and mixed assembly of the obtained clean data. Fragments shorter than 500 bp in all Scaftigs generated from assembly were filtered out. The remaining Scaftigs were used for open reading frame prediction and redundancy filtering for the gene catalog using MetaGeneMark (version: 2.10, http://exon.gatech.edu/GeneMark/meta_gmhmmp.cgi) and CD-HIT software (version: 4.5.8, http://www.bioinformatics.org/cd-hit/). The clean data of each sample were mapped to the gene catalog using Bowtie2.2.4 for further statistical analysis. Taxonomical annotation was performed using the DIAMOND software (Buchfink et al., 2015) to blast the obtained gene catalog against reference sequences from the Non-Redundant Protein Sequence Database (NR database) (version: 2014-10-19) of NCBI. The taxonomic profiling of the organisms in each sample was quantified by MataPhlAn (http://huttenhower.sph.harvard.edu/metaphlan/). DIAMOND software was implemented to blast unigenes against the KEGG and eggnog databases for further functional profiling.

### 2.6 Fecal short-chain fatty detection

Approximately 0.1 g of fecal sample was pretreated with 50 μL of 15% phosphoric acid, 100 mg of glass bead, 100 μL of internal standard solution (125 μg/mL of hexanoic acid), and 400 μL of ether. Then, the mixture was ground at 60 Hz for 60 s in a high-throughput tissue grinder, then centrifuged at 12,000 rpm for 10 min at 4°C. The supernatant was collected for SCFA measurement using a Thermo TRACE 1310-ISQ LT Gas Chromatography-Mass Spectrometry (GC-MS) (Thermo, USA).

### 2.7 Untargeted metabolomics

A total of 0.1 g of fecal samples was individually ground with liquid nitrogen, and the homogenate was resuspended in 400 μL of a methanol and acetonitrile mixture (1:1) by vortexing for 5 min. The samples were incubated at −20°C for 1 h and then centrifuged at 12,000 rpm, 4°C for 15 min. The supernatant was collected and vacuum-dried for further detection. Finally, the supernatant was dissolved in 100 μL of acetonitrile and injected into the liquid chromatography–mass spectrometer (LC-MS) system using the Acquity UPLC Nexera X2 system (SHIMAZDU, JAP, Kyoto Prefecture, Japan) coupled to Triple TOF 5600+ (sciex, USA). Appropriately, 3 μL of the sample was injected onto a ZORBAX Eclipse Plus C18 (3.5 μm * 2.1 mm * 100 mm, Agilent) using a 17-min linear gradient at a flow rate of 0.5 mL/min. Mobile phase A was water containing 0.1% of formic acid, and mobile phase B was acetonitrile containing 0.1% of formic acid. The gradient was set as 2% phase B for 1 min, linearly increased to 90% in 12 min, then a decrease to 2% over the next 1 min, and held at 2% for 4 min. The column was equilibrated for 5 min before every single sample introduction, with the temperature maintained at 40°C. The mass spectrometry was conducted by electrospray ionization in both positive and negative modes, with the following parameters: ion source temperature of 120°C, desorption temperature of 500°C, desolvent nitrogen flow of 600 L/h, cone gas flow of 50 L/h, sampling cone voltage of 27 eV, extraction cone voltage of 4 eV, and a quadrupole scan range of 50–1,500 m/z.

For multivariate analysis, orthogonal partial least-squares discrimination analysis (OPLS-DA) was constructed to determine the distributions and find the metabolic difference using the MetaboAnalyst (http://www.metaboanalyst.ca/MetaboAnalyst/). The variable importance in projection (VIP) scores from OPLS-DA were used for metabolite ranking. The parameters R^2^Y were used to evaluate the fitting condition of the OPLS-DA models, and Q^2^ was used to assess the predictive ability. The volcano plot was used to filter metabolites of interest; the peaks exhibited statistically significant metabolites and the intensity data of these regions were used for further hierarchical cluster and metabolic pathway analysis. For metabolite identification, the assigned modified metabolite ions were confirmed by matching in the Human Metabolome Database (HMDB) (http://www.hmdb.ca/spectra/ms/search) database and KEGG database (http://www.genome,jp/kegg).

### 2.8 Statistical analysis

All quantitative data were presented as the mean ± SEM. Comparison of ACE2 KO vs. WT mice and ACE2-overexpressed vs. GFP mice was tested by the Mann–Whitney *U*-test using GraphPad Prism 8 (GraphPad Software, San Diego, California, USA). The differences were deemed significant at *p* <0.05 and a false discovery rate (FDR) *q*-value <5%. For 16S rRNA and metagenomic sequencing, the bacterial taxa analyses, Venn diagrams, 3D-PCoA, and cluster analyses were carried out using R software (Version 2.15.3). Significant species between groups were assessed using a Kruskal–Wallis (KW) sum-rank test by LEfse. PICRUSt2 was adopted for functional prediction, with statistical significance determined using Welch’s *t*-test with corrections for multiple comparisons via FDR on the STAMP platform. For untargeted metabolomics analysis, the raw data were processed using Mass Spectrometry-Data Independent Analysis software (MS-DIAL) (Tsugawa et al., 2015) and MetaboAnalyst 5.0 (https://www.metaboanalyst.ca/) to obtain clean data, which were counted using the statistical software R (R version R-3.4.3), Python (2.7.6 version), and CentOS (release 6.6). The metabolites with VIP scores>1.0, FC>1.2 or FC<0.833, and *p*-value<0.05 were considered statistically significant contributors. Volcano plots were performed on the basis of log_2_ (fold change) and −log_10_ (*p*-value) of metabolites by ggplot2 in R. The data of differential metabolites were normalized using *z*-scores and plotted by Pheatmap package in R for clustering heatmaps. The metabolic pathway enrichment of differential metabolites was performed using the KEGG database, with *p*-values less than 0.05 considered statistically significant.

## 3 Results

### 3.1 *ACE2* absence alters the microbial community composition

**Figure 1:**
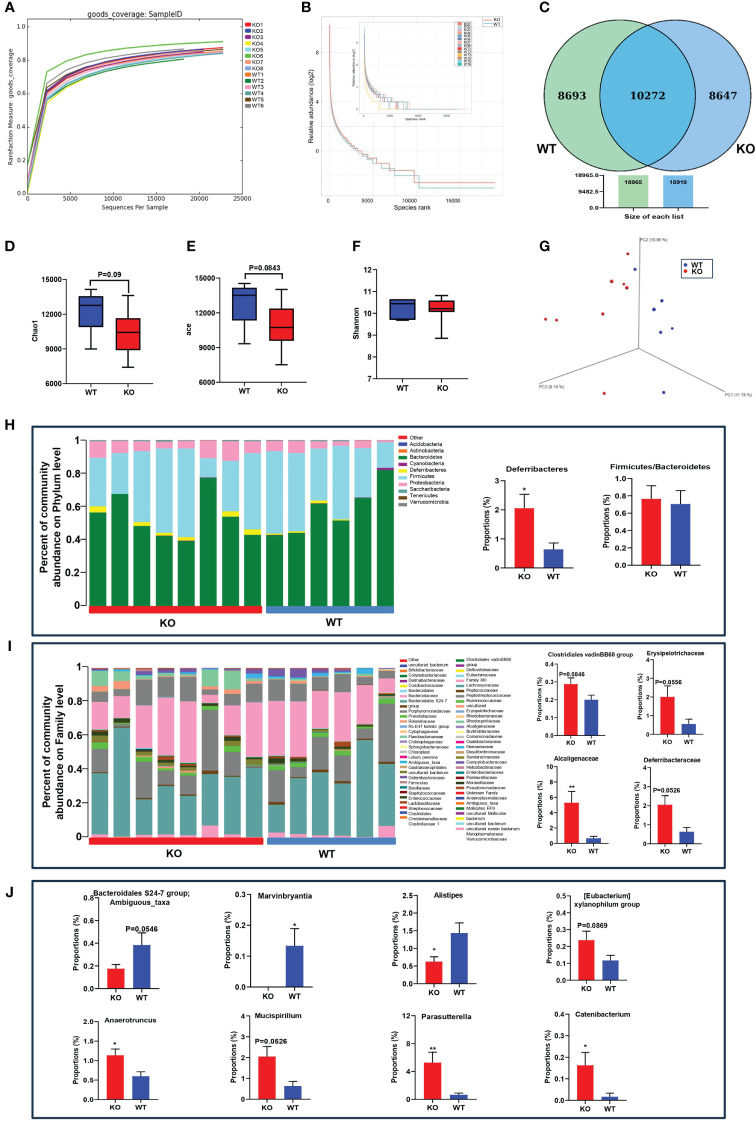
*ACE2* knockout affects the gut microbiome compared with WT mice. **(A)** Rarefaction measure of V3–V4 regions of the16S rRNA gene in gut microbiota. **(B)** The microbial richness and evenness were analyzed by rarefaction curves. **(C)** The differentially abundant bacteria were analyzed by a Venn diagram. **(D–F)** The alpha diversity was evaluated by the Chao1 index, ACE index, and Shannon index. **(G)** The beta diversity was analyzed by 3D-PCoA based on unweighted UniFrac distances. **(H–J)** The gut microbiota constituent profiles at the phylum, family, and genus levels. WT, wild-type mice (*n* = 6); KO and *ACE*2 knockout mice (*n* = 8). Data are presented as mean ± SEM. *p < 0.05, **p < 0.01 vs. WT mice.

To investigate the effects of *ACE2* on gut microbial community structure, we used the *ACE2* knockout mouse model and analyzed the fecal microbiome by sequencing the V3–V4 regions of 16S rRNA. After filtering unqualified tags, we obtained a total of 327,194 reads, with an average of 23,371 ± 5,977 per sample. As sequencing depth increases, the rarefaction index increases sharply with sequences below 5,000, and the rarefaction curve approached a saturation plateau with sequences exceeding 10,000, suggesting comprehensive species coverage in the samples (**Figure 1A**). The rank abundance curves showed similar slopes and widths between the WT and *ACE2* KO group, indicating no significant difference in microbial richness and evenness (**Figure 1B**). According to the abundance of OTUs in each group, the Venn diagram illustrates that a total of 10,272 OTUs were identified as major microbiota, shared by two groups, accounting for approximately 54% of each compartment (**Figure 1C**). For the evaluation of richness and diversity of microbial communities, the alpha diversity values were analyzed. Compared with the WT mice, the *ACE2* KO mice had slightly lower Chao1 and ACE index, but no significant difference in Shannon indexes (**Figures 1D–F**). The microbial community structure (beta diversity) was performed using the 3D-PCoA based on UniFrac distances. As shown in **Figure 1G**, there is an apparent separation between WT and *ACE2* KO groups according to gut bacterial composition.

To clarify the influence of ACE2 in microbial diversity, the obtained bacterial OTUs were further classified according to the species database. The dominant phyla in both groups were *Firmicutes*, *Bacteroidetes*, and *Proteobacteria*. The relative abundance of *Deferribacteres* was significantly increased in the *ACE2* KO mice, considered pathogenic bacteria harmful to the intestinal mucus layer (Ravussin et al., 2012).

The *Firmicutes-*to-*Bacteroidetes* ratio of bacteria abundance, which is linked to obesity, showed no differences between the two groups (**Figure 1H**). At the family level, the proportion of *Alcaligenaceae* increased markedly in *ACE2* KO mice compared to WT mice. The bacteria of *Clostridiales vadinBB60 group, Erysipelotrichaceae*, and *Deferribacteraceae* were also enriched in the *ACE2* KO mice, but these changes were statistically insignificant (**Figure 1I**). At a genus level, *ACE2* deletion substantially decreased the relative abundances of *Marvinbryantia* and *Alistipes*, whereas it increased the relative proportion of *Anaerotruncus, Catenibacterium*, and *Parasutterella*. Moreover, the OTUs corresponding to ambiguous taxa of the *Bacteroidales S24-7* group decreased, whereas those of the Mucispirillum and [Eubacterium] xylanophilum group were enriched in *ACE2* KO mice as compared to the controls (**Figure 1J**). These data collectively demonstrated that the absence of *ACE2* results in the modification of natural taxonomic profiling of the gut microbiota.

### 3.2 *ACE2* overexpression affects the structure and diversity of gut microbiota in obese mice

**Figure 2:**
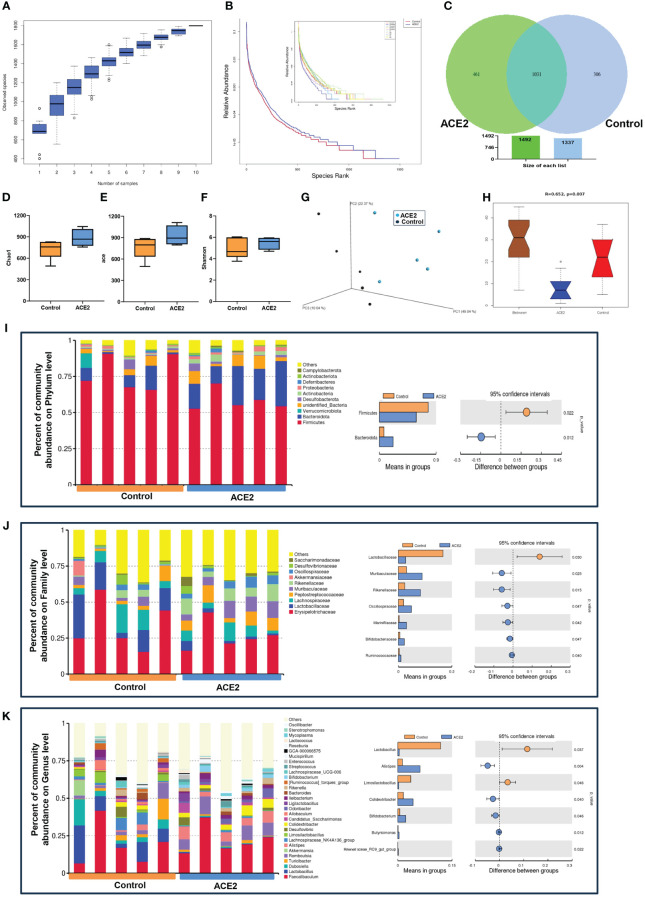
ACE2 overexpression modulates the microbial community in HFD-fed mice. **(A)** The species diversity was detected using the species accumulation boxplot. **(B)** Rank abundance curves. **(C)** Venn diagram. **(D)** Chao1 index. **(E)** ACE index. **(F)** Shannon index. **(G)** Unweighted UniFrac-based 3D-PCoA. **(H)** The significant difference between ACE2 mice and the control group was evaluated by Anosim analysis based on Bray–Curtis distance (*R*-value>0 indicates that the intergroup difference is greater than the intragroup difference; otherwise, the intragroup difference is greater than the intergroup difference). The top 10 or the 30 species with the highest relative abundance at different classifications. **(I)** Phylum level. **(J)** Family level. **(K)** Genus level. Control, Ad-GFP-injected obese mice (*n* = 5) and ACE2, Ad-ACE2-injected obese mice (*n* = 5). Data are presented as mean ± SEM. *p* < 0.05 indicates statistical significance.

We next examined how ACE2 overexpression affects gut microbiota in obese mice; Ad-ACE2 injected mice resulted in *ACE2* upregulation significantly in the liver, but not in the ileum (**Supplementary Figure 1**). On the other hand, 16S rRNA analysis was also conducted in the ACE2-overexpressed obese mice. The species accumulation boxplot (**Figure 2A**) showed that the number of observed species increased continuously, achieving a saturation plateau when the sample size reaches 10, suggesting sufficient sampling for data analysis. The rank abundance curve, used to describe species richness and evenness, suggested that the ACE2-overexpressed group had wider distribution ranges on the horizontal axis and shallower slopes than those in the control group, indicating more abundant and uniform species in the former (**Figure 2B**). In the Venn diagram analysis, there were 1,031 common OTUs shared by the two groups (**Figure 2C**). The ACE2-overexpressed group had more unique OTUs than the control mice (461 vs. 306). For the microbial community richness, Chao1 and ACE indexes in ACE2-overexpressed mice tended to increase, albeit not significantly, while the Shannon diversity index exhibited a similar level between the GFP and ACE2-overexpressed group (**Figures 2D–F**). The 3D-PCoA revealed that the gut microbial communities in the two groups could be clearly separated, with Anosim analysis showing consistent results (**Figures 2G, H**). Collectively, these results suggested the overexpression of ACE2-enriched fecal bacterial diversity.

For the specific compositional changes, the top 10 species with the highest relative abundance at different classifications were analyzed using Perl software. At the phylum level, ACE2-overexpressed mice had a lower abundance of *Firmicutes* and a higher abundance of *Bacteroidetes*, resulting in a decreased *Firmicutes/Bacteroidetes* ratio, which was closely related to weight loss (**Figure 2I**). At the family level, we found a decreased relative abundance of *Lactobacillaceae* and increased richness in *Muribaculaceae*, *Rikenellaceae*, *Oscillospiraceae*, *Marinifilaceae*, *Bifidobacteriaceae*, and *Ruminococcaceae* in the ACE2-overexpressed group compared to the control group (**Figure 2J**). Similarly, there were evident differences between the two groups at the genus level. Compared to the control group, the ACE2-overexpressed group showed a significant increase in the proportion of *Alistipes*, *Colidextribacter*, *Bifidobacterium*, *butyricimonas*, and *Rikenellaceae-RC9-gut group*, while the abundance of *Lactobacillus* and *Limosilactobacillus* decreased (**Figure 2K**).

### 3.3 *ACE2* changes the functions of gut microbiota

**Figure 3:**
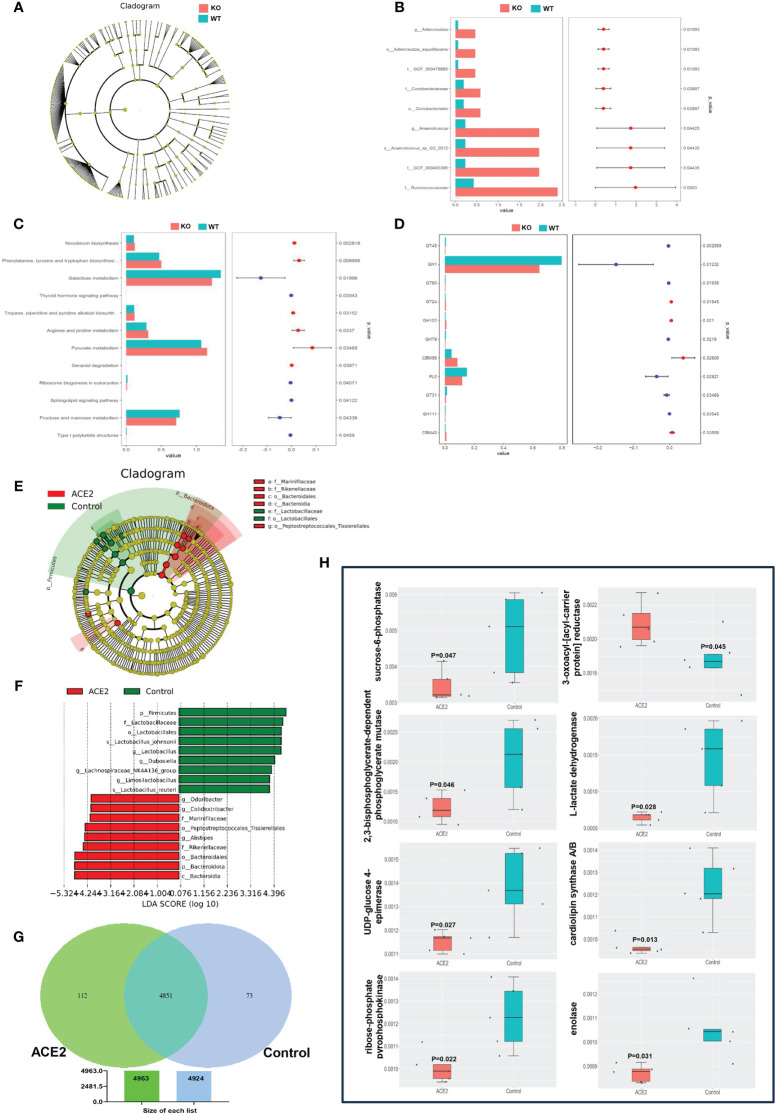
*ACE2* changes the functions of gut microbiota. **(A)** The taxonomic cladogram of the predominant gut microbiota in *ACE2* KO and WT mice. **(B)** The differential microbiota was detected using metagenomic sequencing in *ACE2* KO and WT mice. **(C)** Bar diagram showing significantly different KEGG pathways between *ACE2* KO and WT mice. **(D)** Analysis of functional differences based on the CAZymes database between *ACE2* KO and WT mice. **(E, F)** The intestinal bacterial hierarchy and the most differentially abundant taxon derived from the LEfSe analysis between the control and ACE2 groups. The diameter of each circle is directly proportional to the taxon’s relative abundance. The red nodes represent significantly differential microbial in the ACE2 group, and the green nodes represent the differential biomarkers in the control group. **(G)** The specific and shared genetic information between the control and ACE2 groups was analyzed on the basis of the KEGG ontology (KO) database. **(H)** Bar diagram showing significantly different carbohydrate and lipid metabolism-related gene information between the control and ACE2 groups. WT, wild-type mice (*n* = 6); KO, *ACE*2 knockout mice (*n* = 8); control, Ad-GFP-injected obese mice (*n* = 5); and ACE2, Ad-ACE2-injected obese mice (*n* = 5). Data are presented as mean ± SEM. *p* < 0.05 indicates statistical significance.

Furthermore, the LEfSe method based on linear discriminant analysis (LDA) was employed to identify the most differentially abundant intestinal flora between *ACE2* KO and WT mice. However, the cladogram showed no predominant bacteria between the two groups (**Figure 3A**). To further screen for differential microbiota, we used the deep sequencing shotgun metagenomics. As outlined in **Figure 3B**, *Adlercreutzia_equolifaciens (t:GCF_000478885*) and *Anaerotruncus_sp_G3_2012* (*t:GCF_000403395*), belonging to the *Actinobacteria* phylum and *Firmicutes* phylum, respectively, showed significant abundance increases in *ACE2* KO mice. Moreover, sequencing reads were assigned to the Kyoto Encyclopedia of Genes and Genomes (KEGG) database for functional modules and pathways analysis. A total of 6,040 KEGG modules and 316 KEGG pathways were identified. Twelve pathways showed significant differences between the two groups. Among them, six KEGG pathways related to amino acid and pyruvate metabolism, geraniol degradation, novobiocin biosynthesis, and tropane, piperidine, and pyridine alkaloid biosynthesis were upregulated in *ACE2* KO mice. In contrast, pathways related to galactose, fructose, and mannose metabolism, thyroid hormone and sphingolipid signaling, ribosome biogenesis in eukaryotes, and type I polyketide structures were downregulated compared to the control group (**Figure 3C**). For Cluster of Orthologous Groups of proteins (COG) functional classification, the results showed that only carbohydrate transport and metabolism were statistically significant and enriched in WT mice microbiomes (**Supplementary Figure 2A**). Then, the gene expression profile related to glucose metabolism was analyzed on the basis of the CAZy database, detecting 290 carbohydrate-active enzymes (CAZymes) families. Among them, glycosyl transferases (GTs), glycoside hydrolases (GHs), and carbohydrate esterases (CEs) were the most abundant classes in both groups, but there were no significant changes in the distribution of these CAZymes (**Supplementary Figure 2B**). Meanwhile, 11 CAZymes families exhibited significant differences between the two groups (**Figure 3D**). For the three enzymes with higher abundance, GH1 and polysaccharide lyase family 0 (PL0) were decreased in the *ACE2* KO mice, while CBM56 was increased compared to the control group. These data indicated that the lack of *ACE2* may result in the deregulation of glucose metabolism, particularly affecting the hydrolysis of glycosidic bonds and the decomposition of polysaccharides.

On the flip side, the LEfSe comparison identified 18 discriminative species biomarkers (LDA score>4, *p*<0.05) between ACE2-overexpressed and control groups. Compared to the control group, the ACE2-overexpressed group had an enriched abundance of nine taxa: the phylum *Bacteroidota* and its corresponding class *Bacteroidia*, the order *Bacteroidales*, the families *Rikenellaceae* and *Marinifilaceae*, and the genera *Alistipes*, *Odoribacter*, and *Colidextribacter*, as well as the order *Peptostreptococcales-tissierellales*. Conversely, nine taxa were depleted in the ACE2-overexpressed group: the phylum *Firmicutes* and its corresponding order *Lactobacillales*, the family *Lactobacillaceae*, the genera *Lactobacillus* and *Limosilactobacillus*, the species *Lactobacillus_johnsonii* and *Lactobacillus_reuteri*, and the genera *Dubosiella* and *Lachnospiraceae_NK4A136_group* (**Figures 3E, F**). For the functional prediction using PICRUSt2 based on the KEGG ontology (KO) database, as shown in **Figure 3G**, the ACE2-overexpressed group exhibited more specific genetic information than the control group (112 vs. 73 genes). Additionally, 56 genes showed a significant difference between the ACE2-overexpressed and GFP groups (**Supplementary Figure 3**), with eight genes involved in carbohydrate and lipid metabolism. Specifically, the pathway related to 3-oxoacyl-[acyl-carrier protein] reductase was over-represented in the ACE2-overexpressed group, while pathways for sucrose-6-phosphatase, 2,3-bisphosphoglycerate-dependent phosphoglycerate mutase, L-lactate dehydrogenase, UDP-glucose 4-epimerase, cardiolipin synthase A/B, ribose-phosphate pyrophosphokinase, and enolase were under-represented (**Figure 3H**).

### 3.4 Genetic ablation of *ACE2* in mice influences the fecal SCFA and metabolite levels

**Figure 4:**
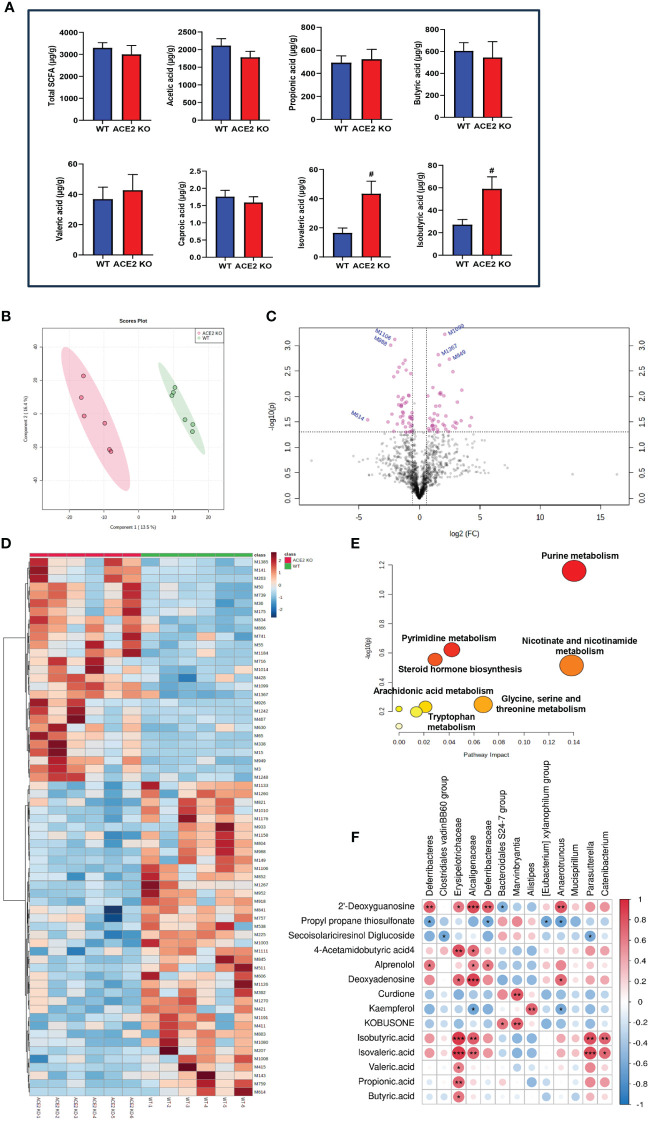
Comparative fecal metabolomic profiles of *ACE2* KO and WT mice. **(A)** Analysis of short-chain fatty acid (SCFA) concentrations in feces by gas chromatography–mass spectrometry (GC-MS). Data are presented as mean ± SEM. #*p* < 0.05 vs. WT mice. **(B)** The PLS-DA score plot of *ACE2* KO (red spots) and WT mice (green spots) in the positive (*R^2^* = 0.939 and *Q^2^* = 0.401). **(C)** Volcano plot [−log10 (*p-*value) and log2 (fold change)] of fecal untargeted metabolomics data in *ACE2* KO and WT mice. Red dots represent false discovery rate (FDR) < 0.05. **(D)** Heatmap of the differential metabolites between *ACE2* KO and WT mice. Red and blue indicate increased and decreased levels, respectively. **(E)** Ingenuity pathway analysis of the 88 differential metabolites based on the KEGG database. The horizontal axis represents the important value of the compound in the pathway, and the vertical axis represents −log10 (*p-*value). The smaller *p-*value and the bigger the pathway impact is, the greater the pathway is influenced. **(F)** Correlations between glucolipid metabolism-related metabolites and differential gut flora in Spearman’s correlation coefficient. The colors range from blue (negative correlation; −1) to red (positive correlation; 1); WT, wild-type mice (*n* = 6); and KO, *ACE*2 knockout mice (*n* = 6). Significant correlations are denoted by *p < 0.05, ** *p* < 0.01, *** *p* < 0.001.

As the major metabolic end products, SCFAs have been reported to play a positive role in improving insulin sensitivity and gut barrier integrity, and reducing fat mass (Cani, 2019). To evaluate how *ACE2* deletion changes the fecal SCFA profile, quantitative analysis of SCFAs was conducted using GC-MS technology. As illustrated in **Figure 4A**, the total SCFA concentrations were similar between *ACE2* KO mice and WT mice. Similarly, there were no differences in the contents of acetic acid, propionic acid, butyric acid, valeric acid, and caproic acid between the two groups. However, a significant increase in branched short-chain fatty acids (BSCFAs), including isovaleric acid and isobutyric acid, was observed in *ACE2* KO mice. These observations confirm that the *ACE2* absence has no effect on the contents of major SCFAs but causes a sharp rise in the levels of BSCFAs, which have been closely associated with the gut environment and glucolipid metabolism homeostasis.

The PLS-DA models were employed to characterize the metabolic differences between classes and the score scatter plot showed a clear separation trend of WT and *ACE2 KO* mice (**Figure 4B**). The cumulative *R^2^* at 0.939 and *Q^2^* at 0.401 for the model indicated the well goodness of fit and predictive ability of the PLS-DA. As shown in the volcano map in **Figure 4C**, a total of 88 differential metabolites that met the criteria with fold changes (FC)>1.5 or <0.667 and *p*<0.05 were identified after the HMDB database screening. The metabolites included carboxylic acids and derivatives, prenol lipids and fatty acyls, nucleosides, and other markers, among which, 43 were upregulated, while 45 were downregulated in *ACE2* KO mice (**Additional File 1**).

Anti-inflammatory factors, such as propyl propane thiosulfonate and secoisolariciresinol diglucoside, and antidiabetic effectors like myricetin, kaempferol, and kobusone were decreased in *ACE2* KO mice compared to WT mice, while harmful factors, such as 2’-deoxyguanosine, 4-acetamidobutyric acid, alprenolol, and deoxyadenosine, were significantly increased. The differential metabolites were further analyzed using hierarchical clustering. The heatmap (**Figure 4D**) showed that the metabolic profiles of *ACE2* KO mice and normal mice were significantly distinguished by clustering.

Moreover, pathway analysis showed that altered metabolites were significantly enriched in seven KEGG pathways (*p* < 0.05, impact > 0.01), namely, purine metabolism; nicotinate and nicotinamide metabolism; glycine, serine, and threonine metabolism; pyrimidine metabolism; steroid hormone biosynthesis; arachidonic acid metabolism; and tryptophan metabolism (**Figure 4E**). The correlations between untargeted fecal metabolites, SCFA, and gut microbiota were analyzed using Spearman’s correlation coefficient (**Figure 4F**). The results showed no statistically significant correlation in acetic acid and caproic acid. Other metabolites and gut microbiota in *ACE2* KO and control groups constitute a dynamic relationship of mutual influence. Furthermore, the metabolites related to glucose and lipid metabolism altered by ACE2 might impact host health. For instance, the BSCFAs (isobutyric acid and isovaleric acid) were positively correlated with the *Erysipelotrichaceae* family (*Catenibacterium* phylum) and the *Alcaligenaceae* family (*Parasutterella* phylum). As a natural flavonoid compound, kaempferol was positively correlated with the *Alistipes* genus, and negatively correlated with the *Alcaligenaceae* and *Anaerotruncus* families.

### 3.5 Overexpression of *ACE2* changes HFD-induced metabolite profiles

**Figure 5:**
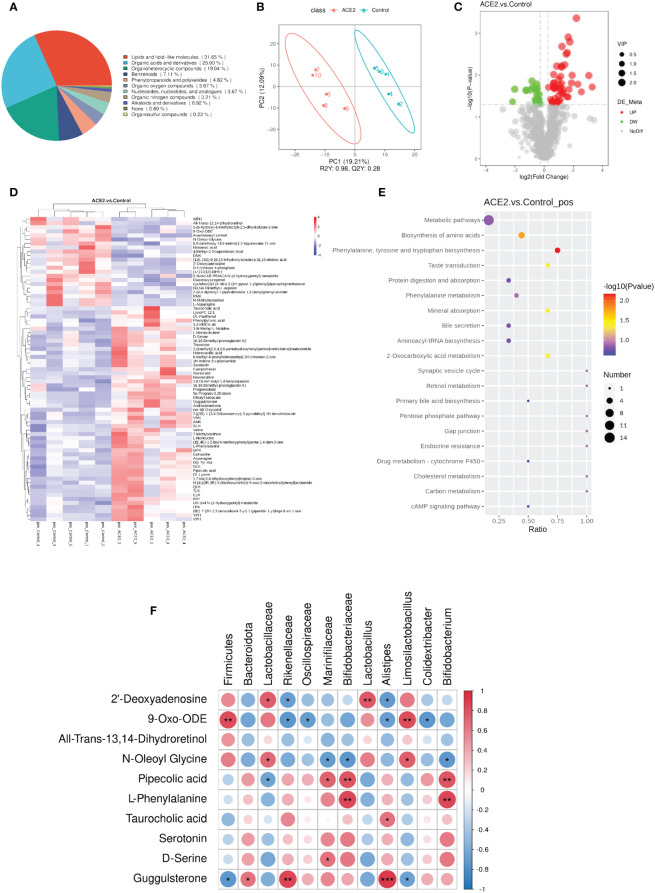
Overexpression of *ACE2* alters HFD-induced metabolite profiles. **(A)** Chemical classification percentage of metabolites identified in the feces of ACE2 and control groups. **(B)** The PLS-DA score plot of ACE2 mice (red spots) and control group (green spots) in the positive (*R^2^* = 0.98 and *Q^2^* = 0.28). **(C)** Volcano plot [−log10 (*p-*value) and log2 (fold change)] of fecal untargeted metabolomics data in ACE2 and control groups. Red dots represent significant upregulated metabolites, and green dots represent significant downregulated metabolites. The size of the dots represents the VIP value. **(D)** Heatmap of the differential metabolites between ACE2 and control groups. Red and blue indicate increased and decreased levels, respectively. **(E)** KEGG pathway enrichment bubble diagram of differential metabolites. The horizontal axis represents the enrichment of the differential compound in the pathway, and the color and size of the dot represent −log10 (*p-*value) and the number of differential metabolites in the corresponding pathway, respectively. **(F)** Correlations between glucolipid metabolism-related metabolites and differential gut flora in Spearman’s correlation coefficient. The colors range from blue (negative correlation; −1) to red (positive correlation; 1). Control, Ad-GFP-injected obese mice (*n* = 5) and ACE2, Ad-ACE2-injected obese mice (*n* = 5). Data are presented as mean ± SEM. Significant correlations are denoted by *p < 0.05, **p < 0.01, ***p < 0.001.

To explore the impact of *ACE2* on fecal metabolites, stool samples from control and Ad-ACE2-treated mice were analyzed using untargeted LC-MS. The chemical classification of identified metabolites showed that the top three categories with the higher proportions were lipids and lipid-like molecules, organic acids and derivatives, and organoheterocyclic compounds (**Figure 5A**). PLS-DA revealed that the fecal metabolic phenotype of Ad-ACE2-treated mice was obviously different from that of control mice (**Figure 5B**). A total of 75 metabolites were significantly altered in the stool of ACE2-overexpressed mice compared to control mice (**Additional File 2**), with 53 metabolites increased and 22 metabolites decreased (**Figures 5C, D**). Furthermore, these dysregulated metabolites were enriched in various metabolomic pathways, with the top 20 KEGG pathways illustrated in **Figure 5E**. Most differential metabolites were enriched in the metabolic pathways and amino acid biosynthesis. Specifically, these altered metabolites were mainly related to lipid and lipid-like molecules, organic acids and derivatives, organoheterocyclic compounds, and purine nucleosides. The potential correlation between differential bacterial species and fecal metabolites related to glucolipid metabolism, inflammation, and intestinal incretin was examined (**Figure 5F**). *Firmicutes* were negatively correlated with guggulsterone and positively correlated with 9-Oxo-ODE. *Bacteroidota* and *Lactobacillaceae* were positively correlated with guggulsterone, 2’-deoxyguanosine, and N-oleoyl glycine, respectively. Significant negative correlations were found between *Rikenellacea* and *Oscillospiraceae* and 9-Oxo-ODE, as well as between *Marinifilaceae* and *Bifidobacteriaceae* and N-Oleoyl Glycine. *Alistipes* showed a positive correlation with guggulsterone and taurocholic acid, and a negative correlation with 9-Oxo-ODE and 2’-deoxyguanosine. Limosilactobacillus showed a positive correlation with N-oleoyl glycine and 9-oxo-ODE, and a negative correlation with guggulsterone. *Colidextribacter* and *Bifidobacterium* were negatively correlated with 9-Oxo-ODE and N-oleoyl glycine, respectively. Interestingly, serotonin and all-trans-13,14-dihydroretinol were not correlated with any species.

## 4 Discussion

Energy metabolism is typically defined as a biological process of energy storage, release, transfer, and utilization during nutrient (mainly glucose, lipid, and proteins) consumption. The energy metabolic perturbation is involved in the development of cardiovascular diseases, neurodegenerative diseases, metabolic diseases, and cancers. ACE2 dysregulation has been revealed to be a major contributing factor to MetS progression, through mechanisms involving inflammation, stress states, structural remolding, and impairment of insulin signaling pathways (Cao et al., 2021). In this study, we have confirmed for the first time that the global ACE2 challenge could alter gut microbiome composition and its metabolites, which were inextricably linked to host energy metabolism. Specifically, we conducted an integrative analysis of metagenomics and metabolomics data in ACE2 knockout and overexpressed mice, as well as in the control subjects. The results showed that ACE2 ablation slightly decreased the microbial community richness, while ACE2 overexpression could restore these effects.

Additionally, the microbial composition and distribution results showed significant differences in both ACE2 KO vs. WT mice and ACE2-overexpressed vs. GFP mice. Furthermore, the expression levels of ACE2 led to complex changes in microbiota function and metabolites, as manifested in the KEGG pathways related to carbohydrate and lipid metabolism, amino acid metabolism, genetic information, and cellular processing. This was also reflected in BSCFA concentrations and other factors related to host energy homeostasis. Taken together, our results provide a potential mechanism for the effects of ACE2 on improving host carbohydrate and fat metabolism.

It is well documented that approximately 100 trillion different microorganisms live in the human intestinal tract, involved in various physiological processes such as digestion, vitamin synthesis, metabolic regulation, and maintenance of the barrier function (Fujisaka et al., 2023). Therefore, disturbance of the enteric micro-ecological environment is related to various diseases. Evidence indicates that multiple factors (e.g., diet, gene, motion, drug, age, hygiene, and host health) can affect the composition and function of gut microbiome (Sankararaman et al., 2022). Our data indicate that the *ACE2* gene plays a significant and beneficial role in gut flora characteristics.

For example, there was a decreased microbiome diversity and richness as well as a higher *Firmicutes/Bacteroidetes* ratio in diabetic and obese individuals (Wang et al., 2022). In this study, we found that *ACE2* KO mice had lower Chao1 (*p* = 0.09) and ACE indexes (*p* = 0.0843), with no differences in the *Firmicutes/Bacteroidetes* ratio (**Figure 1**). In contrast, ACE2 overexpression led to an upward trend in microbiome richness, but an obvious decreased *Firmicutes/Bacteroidetes* ratio (**Figure 2**).

Moreover, the abundance and proportion of the phylum *Deferribacteres* and its corresponding family *Deferribacteraceae* and the genus *Mucispirillum* are significantly higher in *ACE2* KO mice, which have also been reported in obese mice. These bacterial species are considered conditional pathogens associated with intestinal inflammation (Ravussin et al., 2012; Herp et al., 2021). A large body of evidence underlines that the family *Alcaligenaceae* is negatively correlated with acetic acid (one of the main components of SCFA), and its phylum *Parasutterella* contributes to intestinal chronic inflammation (Chen et al., 2018; Lu et al., 2022). The *Erysipelotrichaceae* family and its *Catenibacterium* genus are enriched in obese subjects and patients with MetS with associated T2DM and are also related to hepatic steatosis progression (Spencer et al., 2011; Gradisteanu Pircalabioru et al., 2022). The genera *Anaerotruncus* and *[Eubacterium] xylanophilum group*, belonging to the *Clostridiales* order, are harmful to the intestinal barrier due to the increased LPS production (Gao et al., 2020). Interestingly, these bacterial species are found to have a significant increase in ACE2 KO mice.

Furthermore, an increase in the relative abundance of *Anaerotruncus_sp_G3_2012* and *Adlercreutzia_equolifaciens* was found in ACE2 KO mice. The former had been reported to be closely associated with the pathological status of diabetic *db/db* mice (Zhang et al., 2022), while the latter was well described as an equol-producing species that exerts lipid-lowering effects (Zheng et al., 2019), potentially serving as a compensatory mechanism for the metabolic disorders of *ACE2* KO mice. In addition, ACE2 KO mice experienced an increased relative abundance of the *Clostridiales vadinBB60 group*, considered an SCFA-producing microbiome and positively correlated with the proportion of propionate, which may stimulate intestinal gluconeogenesis (Tirosh et al., 2019). In contrast, our study indicated that ACE2 KO mice had a significant decrease in the abundance of *Bacteroidales S24-7 group_norank*, *Marvinbryantia*, and *Alistipes*, which are part of the gut microbiota core and among the main SCFA producers. These bacteria were involved in the production of butyrate and acetate, which could prevent IR and overactive inflammation while maintaining intestinal homeostasis (Parker et al., 2020; Xiao et al., 2020; Guo et al., 2022).

Consistently, previous evidence points to a direct role for the ACE2 mutation in reshaping gut microbial ecology, inducing intestinal inflammation, and causing diarrheal processes, along with tryptophan malnutrition, impaired expression of antimicrobial peptides, and changes in the ACE2/mTOR/autophagy pathway (Hashimoto et al., 2012; Duan et al., 2019; de Olieveira et al., 2020). Considering this, it is necessary to test the influence of ACE2 upregulation in gut microbiome signatures (**Figures 2**, **3**). The data presented here suggested that certain SCFA-producing microbes, such as *f-Rikenellaceae* (*g-Alistipes* and *g-*Rikenellaceae-*RC9-gut group*), *f-Muribaculaceae* (also known as *Bacteroidales* S24-7), *f-Ruminococcaceae*, and *g-Odoribacter*, were significantly increased in ACE2-overexpressed mice. In contrast, *Alistipes* and *Bacteroidales* S24-7 C were depleted in *ACE2* KO mice. Moreover, ACE2 overexpression increased the abundance of *f-Oscillospiraceae* (*g-Colidextribacter*), *f-Marinifilaceae* (*g-butyricimonas*), and *f-Bifidobacteriaceae* (*g-Bifidobacterium*), which were considered anti-inflammatory probiotic beneficial for glucose tolerance and lipid disorders via stimulating secondary bile acid (BA) production and GLP-1R pathway (Gurung et al., 2020; Lee et al., 2022; Zhang et al., 2023). On the other hand, there was an enrichment of the *f-Lactobacillaceae* (*g-Lactobacillus* and *g-Limosilactobacillus*), *f-Erysipelotrichaceae* (*g-Dubosiella*), and *f-Lachnospiraceae* (*Lachnospiraceae_NK4A136_group*) in the control obese mice compared to ACE2-overexpressed mice. This is consistent with findings in the T2DM cohort and mice, suggesting a positive correlation between these species and blood glucose levels, liver injury, lipid metabolism, and ROS (Karlsson et al., 2013; Wang et al., 2020; Chen et al., 2023). However, the function of these bacteria may not be constant. Recent evidence has shown that they can improve fasting blood glucose (FBG), reduce inflammation, enhance intestinal permeability, and promote PI3K/AKT signaling pathway (Hu et al., 2019; Lacerda et al., 2022; Li et al., 2023). Further investigation is needed to fully understand their implications.

Given the important role of ACE2 challenge in microbial community composition and energy homeostasis, we further investigated the predicted functions of gut microbiota (**Figure 3**), particularly in glucose and lipid metabolism. Interestingly, the predicted functional compositions of galactose, fructose, and mannose metabolism, along with the sphingolipid signaling pathway, were significantly downregulated by *ACE2* deficiency. This downregulation was accompanied by decreased levels of GH1 and PL0, resulting in the increase of carbohydrate levels in feces and sphingolipid accumulation, which may cause IR (Zywno et al., 2021; Takeuchi et al., 2023). Conversely, amino acid and pyruvate metabolism were compensatory upregulated in *ACE2* KO mice. Amino acid malnutrition caused by the absence of ACE2, coupled with B0AT1 (namely SLC6A19, solute carrier family 6 (neutral amino acid transporter), member 19), may reactively stimulate amino acid metabolism. Pyruvate metabolism is considered one of the main pathways for non-absorbed carbohydrate metabolism. resulting in SCFA production. On the other hand, genetic information from the gut microbiota of ACE2-overexpressed mice showed a close correlation with fatty acid, sucrose, and cardiolipin biosynthesis, galactose degradation, glycolysis, and gluconeogenesis. Overall, these findings indicate that the regulatory effect of ACE2 on host glucolipid metabolism may partially be attributed to the alteration of intestinal flora.

As the direct energy source of intestinal mucosal cells, SCFAs strongly influence host energy metabolism by regulating the release of gut hormones (incretin GLP-1, PYY, and GLP-2), sympathetic activity, and glucolipid metabolism in peripheral tissues (Gurung et al., 2020; Cunningham et al., 2021). Specifically, acetate, propionate, and butyrate are the most abundant SCFAs, which enhance the function of islet β-cells; increase fatty acid oxidation and insulin sensitivity in liver, muscle, and adipose tissue; promote thermogenesis; and suppress fatty acid synthesis. They achieve these effects by coupling with G-protein-coupled receptors (GRP43, GRP119, and GRP41), inhibiting histone deacetylases and NF-κB/TNFα signaling, and activating the AMPK pathway, among other mechanisms (He et al., 2020; Cunningham et al., 2021). In this study, the absence of ACE2 did not change the content of total SCFAs or the levels of these three main components. This can be attributed to the enrichment of the *Clostridiales vadinBB60 group*, *Mucispirillum*, and *[Eubacterium] xylanophilum group* in *ACE2* KO mice. Unlike SCFAs, the BSCFAs, such as isovaleric acid and isobutyric acid, are generated from aliphatic amino acids’ catabolism (valine and leucine), which had been proven to be higher in subjects with hypercholesterolemia, associated with an unfavorable lipid profile (Granado-Serrano et al., 2019). It is worth noting that BSCFAs were significantly higher in *ACE2* KO mice than in the control group, which could be partially explained by the differences in bacterial relative abundance and the upregulated amino acid metabolism (**Figure 4**). In turn, the increased proteolytic fermentation may strengthen the accumulation of indole, amines, p-cresol, or ammonia among other compounds, which could aggravate inflammation and local disease states (Hughes et al., 2000).

Chronic and systemic inflammation has been considered as an inducer of diabetes and its complications (Rohm et al., 2022). Apart from SCFAs, ACE2 also altered the production of other metabolites that participated in host inflammatory states and energy metabolism. A growing body of evidence suggests that curdione (Zhou et al., 2017), equol (Akahane et al., 2021), kobusone (Choi et al., 2021), kaempferol (Yang et al., 2022), and myricetin (Song et al., 2021) exert anti-diabetic and anti-lipotoxic effects through enhancing insulin secretion and sensitivity, improving Akt activity, stimulating GLP-1 action and glucose consumption, and suppressing adipogenesis and pancreatic beta-amyloidosis. Of interest, these metabolites and anti-inflammatory factors (propyl propane thiosulfonate and secoisolariciresinol diglucoside) decreased sharply with ACE2 absence, while the insulin-release inhibitory factors (alprenolol, deoxyadenosine, and 2’-deoxyguanosine) significantly increased (Holm et al., 1980; Parikh et al., 2019; Vezza et al., 2021; Ngamjariyawat et al., 2023). Among these, 2’-deoxyguanosine, which is part of the purine metabolism pathway, was consistent with the finding from KEGG pathway analysis. Furthermore, the 4-acetamidobutyric acid, a potential early biomarker for diabetic kidney disease, was upregulated in ACE2 KO mice, which was positively correlated with the families of *Erysipelotrichaceae* and *Alcaligenaceae* (Pan et al., 2022). Unlike the fecal metabolomics results of ACE2 KO mice, ACE2 overexpression significantly reduced the concentration of 2’-deoxyguanosine in HFD-fed mice, as well as the all-trans-13,14-dihydroretinol, a part of retinol metabolism, which was previously reported to be closely related to MetS in adults (Wei et al., 2023).

We further found that microbial metabolites, including 9-Oxo-ODE and N-oleoyl glycine, were lowered in the ACE2-overexpressed group. These metabolites have been identified as MetS-linked metabolites involved with lipid accumulation and reduced gut bacterial load (Wang et al., 2015; Tan et al., 2023). Importantly, the metabolites, such as pipecolic acid, L-phenylalanine, taurocholic acid, serotonin, D-serine, and guggulsterone, have been proven to have anti-inflammatory actions and possess anorexic and hypoglycemic properties (Lam and Heisler, 2007; Yamada and Sugimoto, 2016; Cheng et al., 2018; Nishitani et al., 2018; Lockridge et al., 2021; Osuga et al., 2022). Elevated levels of these metabolites in ACE2-overexpressed mice might suppress the appetite and lipid deposition and enhance GLP-1 secretion, resulting in restored glucose and lipid metabolism disorder. These results jointly indicate the important roles of these changed metabolites in the beneficial effects associated with ACE2.

In summary, we (and other studies) found that *ACE2* deficiency could disrupt the gut-vascular integrity, increase the number of conditional pathogens, and decrease the anti-inflammatory metabolites. The impaired intestinal barrier function and increased conditional pathogens caused by ACE2 deficiency can ultimately lead to the bacteria entering the bloodstream and migrating to peripheral tissues, and result in immune system impairment and local tissues’ inflammatory response, and consequential loss of function (e.g., islet β-cell dysfunction, IR, and fatty liver disease). In turn, hyperglycemia exacerbates immune system disorder and reshapes gut microbiota structure and composition. However, ACE2 overexpression could reverse these effects by increasing anti-inflammatory probiotic and its metabolites. On the other hand, ACE2 overexpression activates the GLP-1R pathway through related beneficial bacteria and metabolites, which could directly promote insulin secretion and improve glucose tolerance and lipid disorders.

## 5 Conclusions

Overall, our findings identified a significant association between ACE2 status and gut microbiome and metabolome profiles, providing a novel mechanism for understanding the positive effects of ACE2 on energy homeostasis. This effect may be due to the optimization of gut microbiota structure and function and the beneficial role of fecal metabolites. However, the specific mechanisms of gut microbiota dysbiosis in ACE2 knockout mice and how the altered intestinal flora combined with its metabolites regulates glucose and lipid metabolism has not been explored in depth. Thus, further studies using an intestinal epithelium-specific ACE2 deletion model and microbiota transplantation would be required. Additionally, factors such as intestinal barrier function, host inflammation, and incretin hormone need to be considered.

## Data availability statement

The data analyzed in this study is subject to the following licenses/restrictions: The original contributions presented in the study are included in the article/**Supplementary Materials**. Further inquiries can be directed to the corresponding authors. Requests to access these datasets should be directed to XC, xicao@ccmu.edu.cn.

## Ethics statement

The animal study was reviewed and approved by the Ethics Committee of Animal Research at Beijing Tongren Hospital, Capital Medical University, Beijing, China (TRLAWEC2022-16). The study was conducted in accordance with the local legislation and institutional requirements.

## Author contributions

LS: Data curation, Funding acquisition, Methodology, Writing – original draft. WJ: Methodology, Writing – review & editing. XC: Conceptualization, Funding acquisition, Methodology, Project administration, Writing – original draft, Writing – review & editing.

## Supplementary material

Supplementary Figure 1 Predicted metabolic functions of gut microbiota in *ACE2* KO and WT mice. **(A)** Bar diagram showing significantly different COG pathways between *ACE2* KO and WT mice. **(B)** The relative abundance of carbohydrate-active enzyme genes in gut microbiota based on the CAZy database. WT, wild-type mice (*n* = 6); KO, *ACE*2 knockout mice (*n* = 8); AA, Auxiliary Activities; CBM, carbohydrate-binding module; CE, carbohydrate esterases; GH, glycoside hydrolase; GT, glycosyltransferase; PL, polysaccharide lyase family; Data are presented as mean ± SEM. *p* < 0.05 indicates statistical significance.

Supplementary Figure 2 Predicted functional differences of gut microbiota in Ad-ACE2- and Ad-GFP-treated mice. **(A)** mRNA expression levels of *ACE2* genes in the liver and ileum of ACE2 and control groups. **(B)** The predicted functional differences in gut microbiota of ACE2 and control groups based on the KEGG database. Control, Ad-GFP-injected obese mice (*n* = 5) and ACE2, Ad-ACE2-injected obese mice (*n* = 5). Data are presented as mean ± SD. *p* < 0.05 indicates statistical significance.

Additional File 1 Information on regulated differentially fecal metabolites between the *ACE2* KO and WT mice based on metabolomic profile.

Additional File 2 Details of the 75 fecal metabolites between the ACE2 and control groups.
