# Supplementary figures and images for: Integrated analysis of gut microbiome and its metabolites in ACE2-knockout and ACE2-overexpressed mice
Source: Front Cell Infect Microbiol. 2024 Jul 17;14:1404678. doi: 10.3389/fcimb.2024.1404678 (PMC11288824; doi:10.3389/fcimb.2024.1404678)

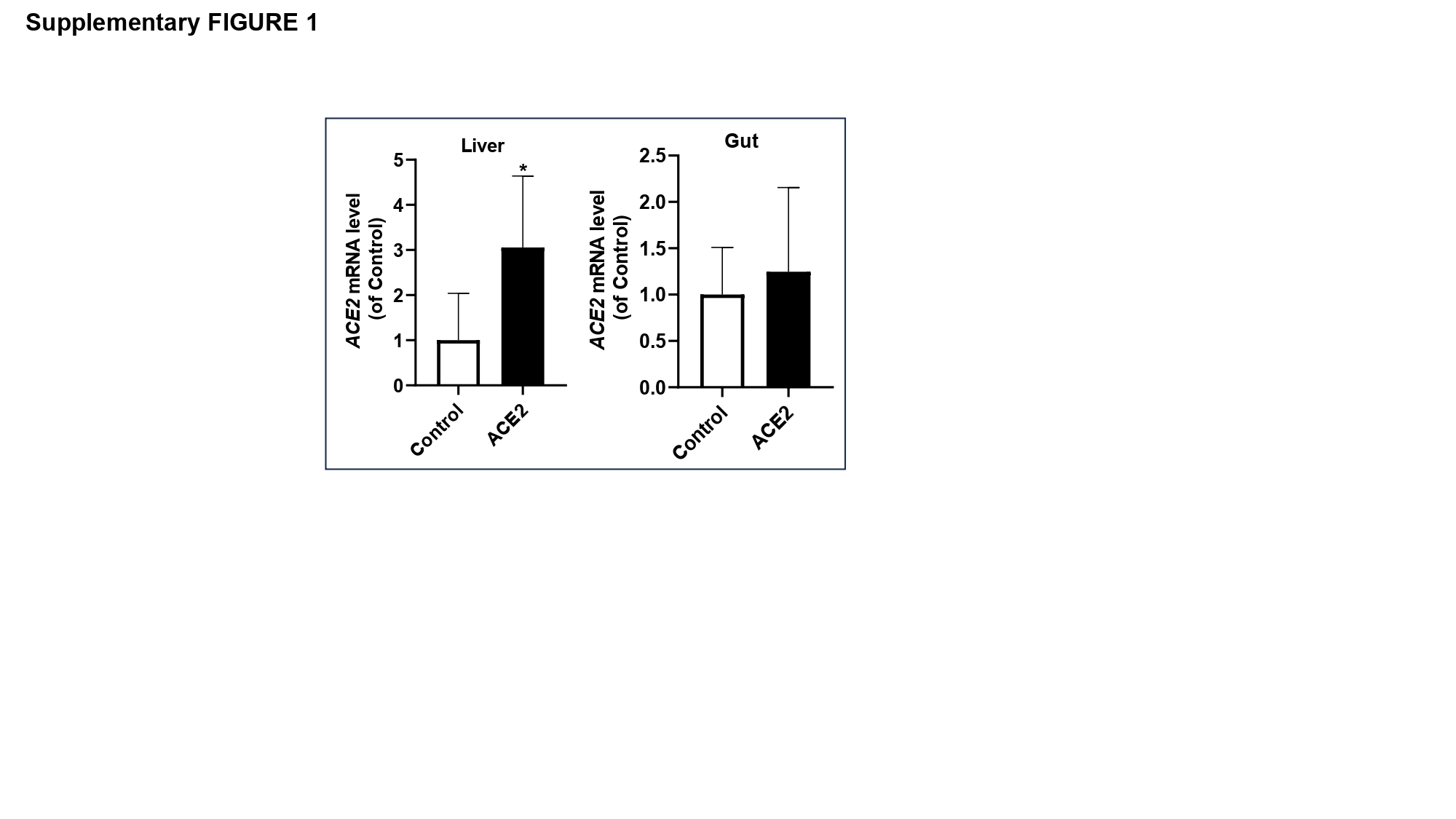

Supplement: Supplementary Figure 1 — Predicted metabolic functions of gut microbiota in ACE2 KO and WT mice. (A) Bar diagram showing significantly different COG pathways between ACE2 KO and WT mice. (B) The relative abundance of carbohydrate-active enzyme genes in gut microbiota based on the CAZy database. WT, wild-type mice (n = 6); KO, ACE2 knockout mice (n = 8); AA, Auxiliary Activities; CBM, carbohydrate-binding module; CE, carbohydrate esterases; GH, glycoside hydrolase; GT, glycosyltransferase; PL, polysaccharide lyase family; Data are presented as mean ± SEM. p < 0.05 indicates statistical significance. [file Image_1.jpg]

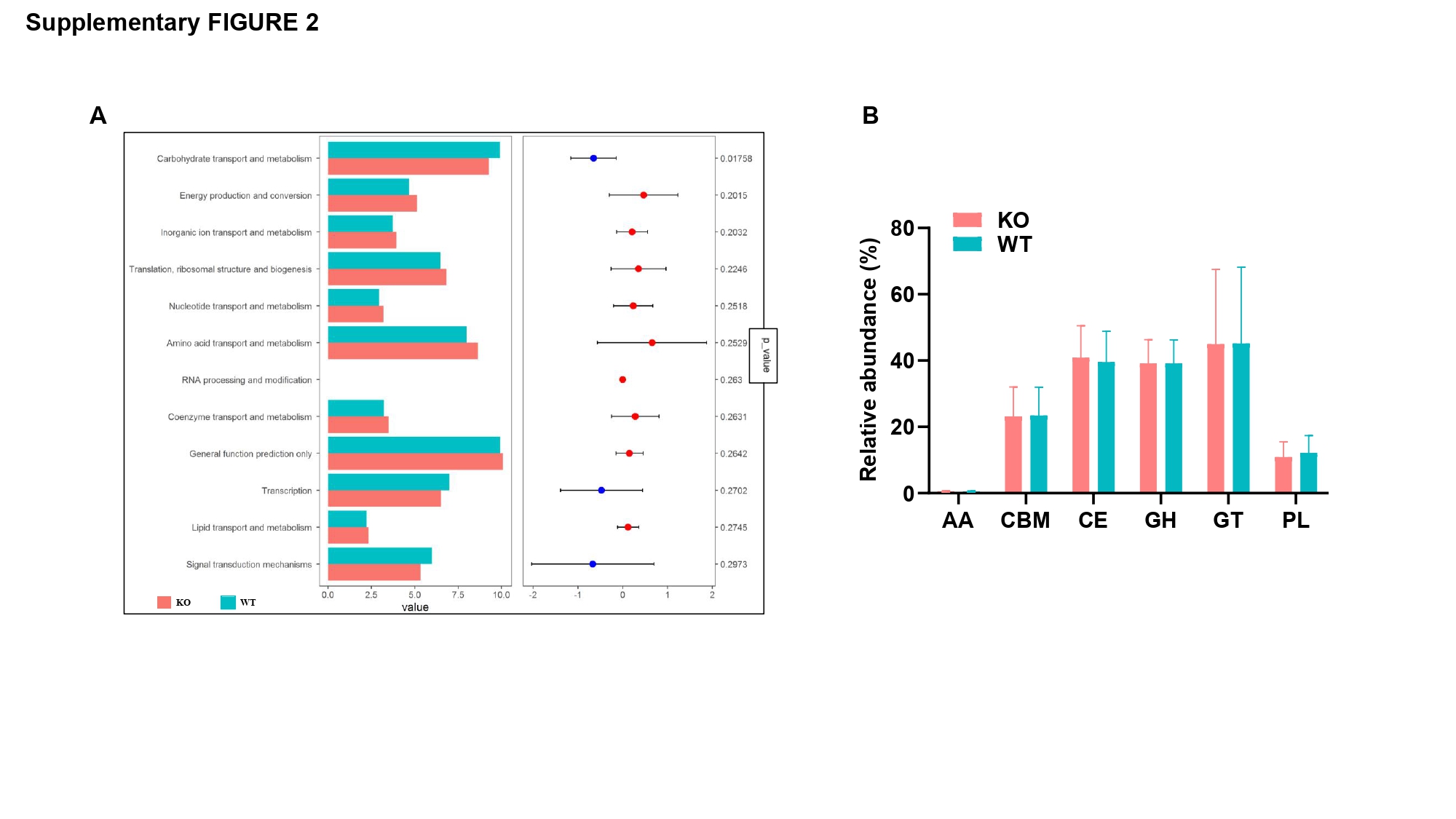

Supplement: Supplementary Figure 2 — Predicted functional differences of gut microbiota in Ad-ACE2- and Ad-GFP-treated mice. (A) mRNA expression levels of ACE2 genes in the liver and ileum of ACE2 and control groups. (B) The predicted functional differences in gut microbiota of ACE2 and control groups based on the KEGG database. Control, Ad-GFP-injected obese mice (n = 5) and ACE2, Ad-ACE2-injected obese mice (n = 5). Data are presented as mean ± SD. p < 0.05 indicates statistical significance. [file Image_2.jpg]

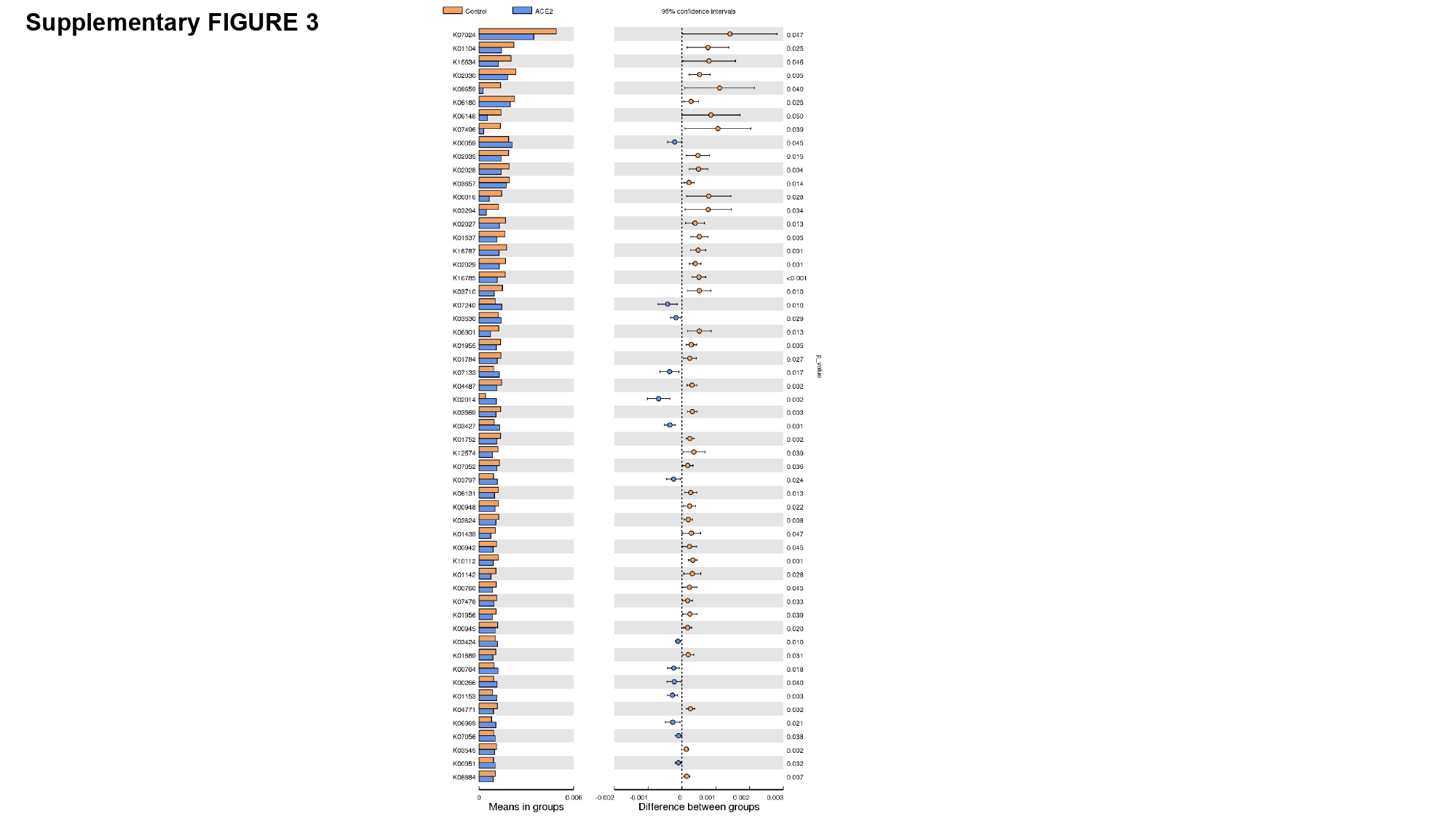

Supplement: Supplementary file 3 [file Image_3.jpg]
